# Supplementary material for: Introducing carbon assimilation in yeasts using photosynthetic directed endosymbiosis
Source: Nat Commun. 2024 Jul 16;15:5947. doi: 10.1038/s41467-024-49585-3 (PMC11252298; doi:10.1038/s41467-024-49585-3)
Supplement: Supplementary file 1 — Supplementary Information [file 41467_2024_49585_MOESM1_ESM.pdf]

Introducing carbon assimilation in yeasts using photosynthetic directed endosymbiosis

Gao *et al.*

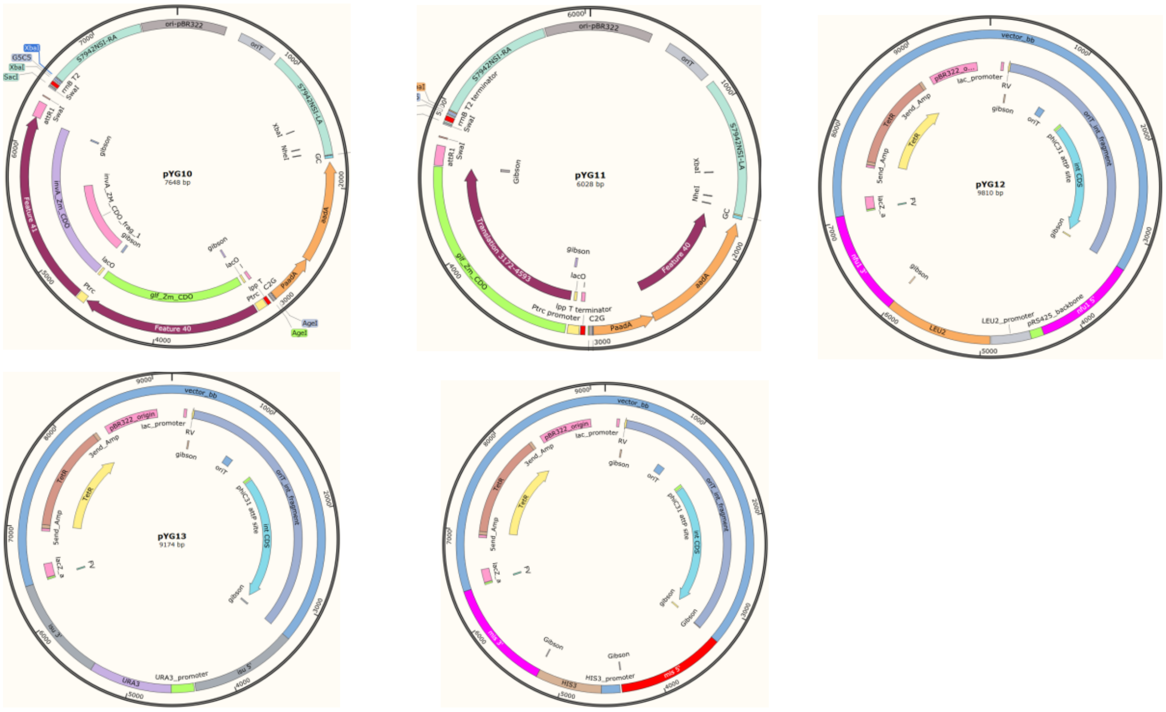

Supplementary Figure 1. Plasmids used in this study. *glf* gene encodes for a glucose facilitator gene that acts as a glucose transporter that is able to secrete glucose upon build up and the *invA* gene encodes for an intracellular invertase that breaks down sucrose into glucose and fructose; CmR – chloramphenicol acetyltransferase; KanR – aminoglycoside-3'-phosphotransferase; *aadA* - streptomycin 3'-adenylyltransferase; *TetR* - tetracycline resistance cassette*; trc, tpi1,* and *pgk1* are constitutive promoters.

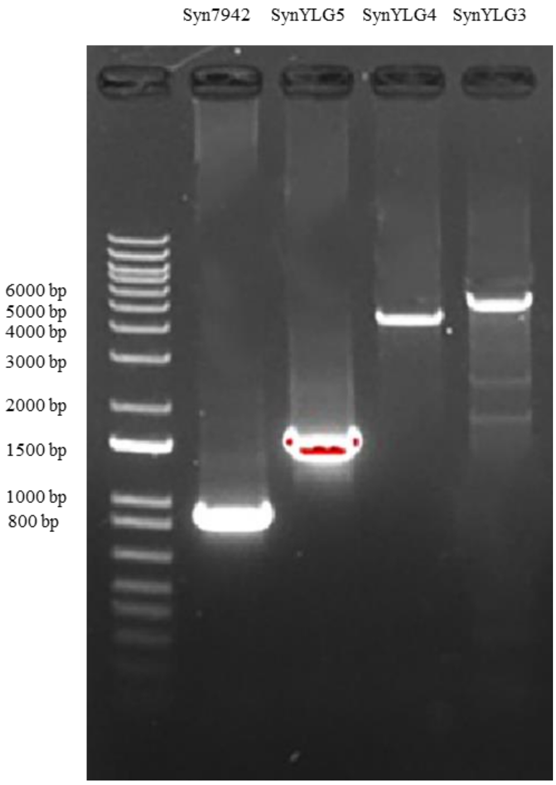

Supplementary Figure 2. PCR amplification of the NSI locus of Syn7942 to confirm recombination. Recombination was verified by DNA sequencing analysis of amplified and gel purified DNA fragments. The experiment was repeated twice independently with similar results. Source data are provided as a Source Data file.

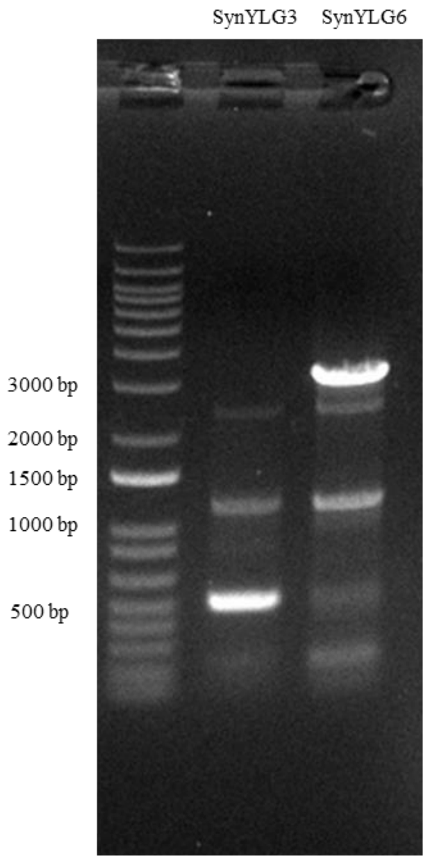

Supplementary Figure 3. PCR amplification of the NSII locus of Syn7942 to confirm recombination. Recombination was verified by DNA sequencing analysis of amplified and gel purified DNA fragments. The experiment was repeated twice independently with similar results. Source data are provided as a Source Data file.

A **B** **C**

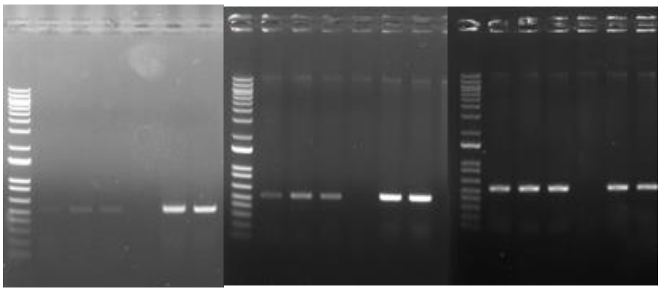

Supplementary Figure 4. PCR amplification of the MAT and CAT locus of fusion yeast to confirm chimera. A, B and C correspond to genomic DNA analysis by PCR for total DNA isolated from yeast/cyanobacteria chimera grown in rounds 3, 4, and 5 of repropagation under selection conditions. In each cases, Line 1: 1 kb DNA ladder; line 2: *S. cerevisiae cox2-60* MAT**a** gene, line 3: *S. cerevisiae cox2-60*:SynJEC3 MAT**a** gene, line 4: *S. cerevisiae cox2-60*:SynYLG6 MAT**a** gene; line 5: *S. cerevisiae cox2-60* CAT gene, line 6: *S. cerevisiae cox2-60*:SynJEC3 CAT gene, line 7: *S. cerevisiae cox2-60*:SynYLG6 CAT gene. Source data are provided as a Source Data file.

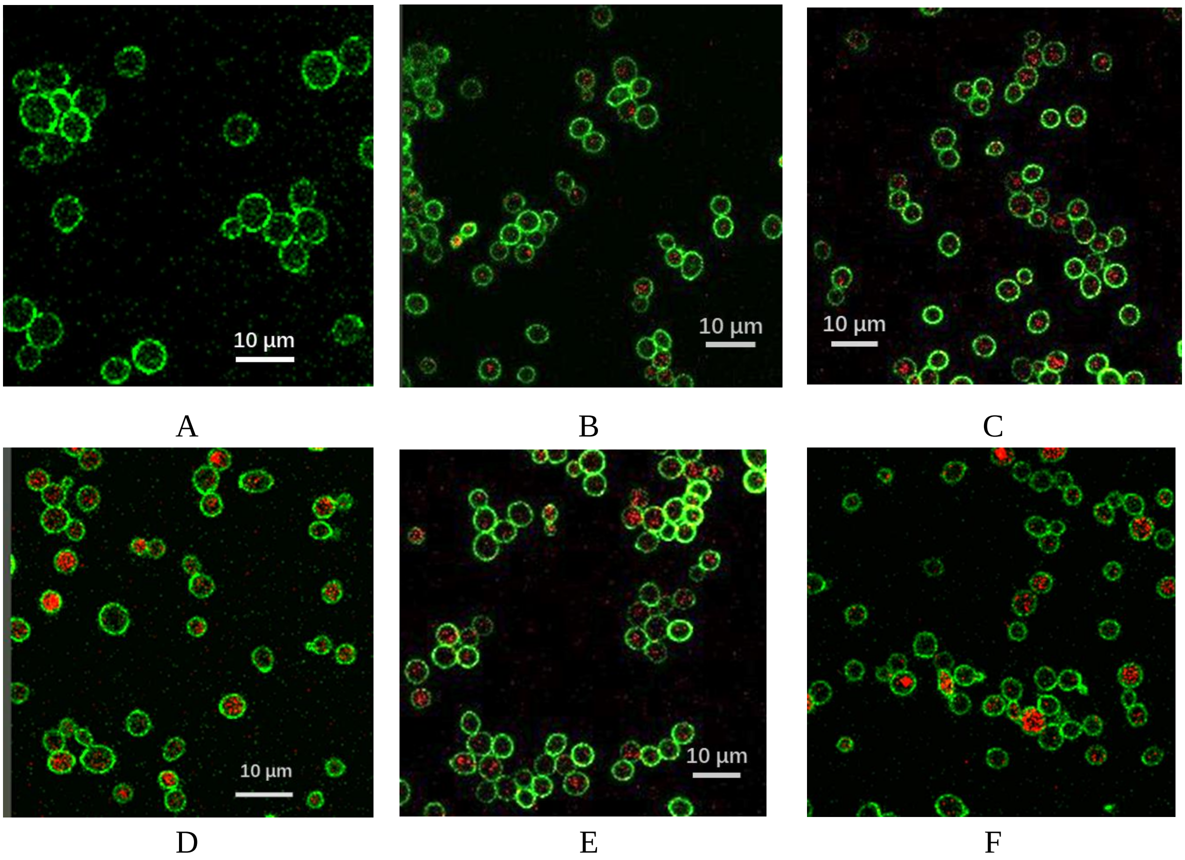

Supplementary Figure 5. Tracking the yeast/cyanobacteria chimeras during various stages of selection using fluorescence confocal microscopy. (A) Control *S. cerevisiae cox2-60* stained with Con A-FITC. (B)-(F) Images of early stages to late stages Yeast/SynYLG6 chimeras propagated for multiple rounds of selection imaged by fluorescence confocal microscopy. The yeasts were stained with Con A-FITC (pseudo-color: green, Ex. = 488 nm; Em. = 510/20) and cyanobacterial signals were monitored by cyanobacterial fluorescence (pseudo-color: red, Ex. = 561 nm; Em. = 650/20). As we go from round 2 to round 3 under strict selection conditions, we see 30 to 60% of the yeast cells containing cyanobacteria and under these conditions, we observe that the cyanobacterial numbers can vary from 0 to 2. The experiment was repeated twice independently (N=2) with similar results.

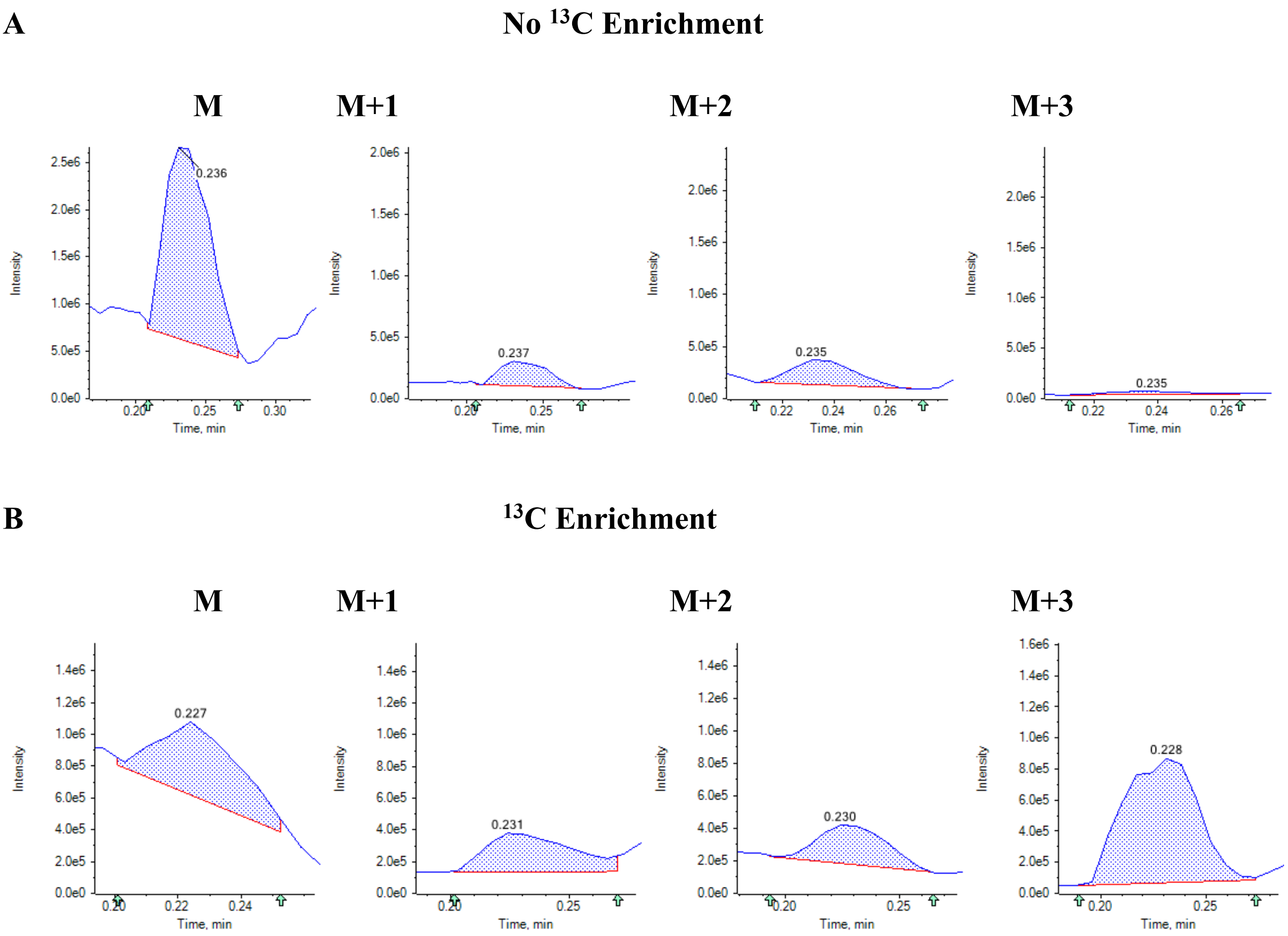

Supplementary Figure 6. Extracted ion chromatograms (EIC) for PEP and its charged states in samples having no ^{13}C enrichment and those detecting ^{13}C enrichment. (A) EIC for no ^{13}C enrichment sample and (B) ^{13}C enrichment sample are displayed. Targeted LC-MS analysis of unlabeled PEP (M) was measured at *m/z* 169, as well as ^{13}C-labeled PEP isotopes of the precursor measured at three charged states: M+1 (M+^{13}C, *m/z* 170), M+2 (M+2^{13}C, *m/z* 171), and M+3 (M+3^{13}C, *m/z* 172). Intensity is measured as peak area. Figures generated in Sciex MultiQuant 3.1 software. Source data are provided as a Source Data file.

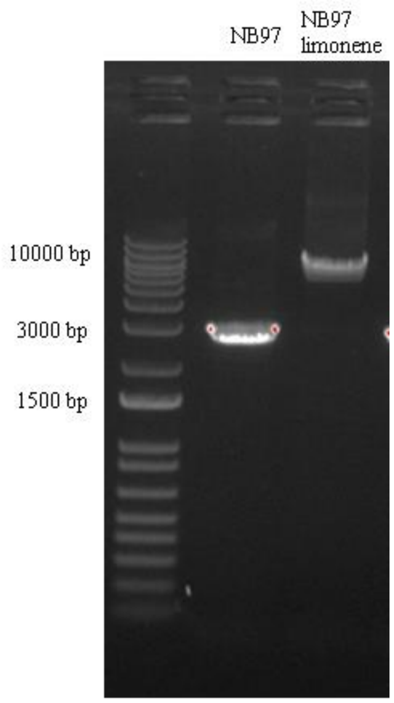

Supplementary Figure 7. PCR amplification to confirm NB97 limonene recombination. Recombination was verified by DNA sequencing analysis of amplified and gel purified DNA fragments. The experiment was repeated twice independently with similar results. Source data are provided as a Source Data file.

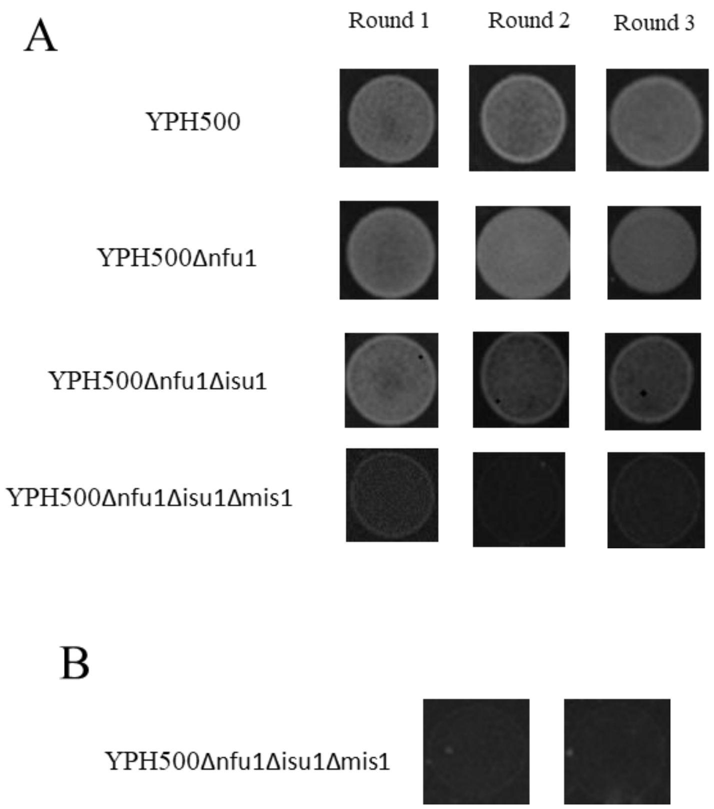

Supplementary Figure 8. YPH500 and mutants grow in YPG and Selection III medium. (A) YPH500 and mutants grow in YPG medium (2% peptone, 1% yeast extract, 2% glycerol) for 3 rounds. (B) YPH500ΔnfuΔisuΔmis grows in Selection III medium.

Supplementary Table 1. The doubling detected for all yeast/cyanobacteria chimera under carbon assimilating selection conditions.

**Yeast/cyanobacteria chimera**	**Total doubling detected**
S. cerevisiae cox2-60	2
S. cerevisiae cox2-60-SynJEC3	3
S. cerevisiae cox2-60-SynYLG6	22
S. cerevisiae YPH500ΔnfuΔisuΔmis	5
S. cerevisiae YPH500ΔnfuΔisuΔmis-SynJEC3	15
S. cerevisiae YPH500ΔnfuΔisuΔmis-SynYLG6	25

Supplementary Table 2. All links of plasmids in this paper.

**Plasmid**	**Link**
pYG10	https://benchling.com/s/seq-NOKzTzfqxoTDdd3fkSRC?m=slm-Neub6NevPaSHHqx3ExQu
pYG11	https://benchling.com/s/seq-D9S24xmf5KaoD9Y3FDrE?m=slm-QRqsl3A1R3eWeRbtWmhA
pYG12	https://benchling.com/s/seq-2b2HZK3SrdlIK2Bp7s64?m=slm-VRHDgXEWsBlLtg04pKHF
pYG13	https://benchling.com/s/seq-GX8hAQhUqDCwXPvhdK0w?m=slm-Kb5fgenTcgJ2TrQIL13d
pYG14	https://benchling.com/s/seq-kMf6DJQTPxMhH37ka7Uz?m=slm-3bMBtd60h9p3z33HoRAA
pYG15	https://benchling.com/s/seq-2FqvOZIrdhuRht6mdQ1b?m=slm-nGfuXsnDuP4onSckdxqV
pYG16	https://benchling.com/s/seq-fcvYj0skUdTxop2IHCRd?m=slm-7XrInpKuIgEIBPcrwJjc
pETLuciferase	https://benchling.com/s/seq-H21UdVUYRnCZiNcFUOS2/edit

Supplementary Table 3. Mass spectrometer parameters for measurement of PEP.

Metabolite	Mass	Q1	Q3	CE	DP	EP	CXP
PEP	M	169	169	5	75	10	10
PEP isotope	M+1	170	170	5	75	10	10
PEP isotope	M+2	171	171	5	75	10	10
PEP isotope	M+3	172	172	5	75	10	10
