# Supplementary material for: Introducing carbon assimilation in yeasts using photosynthetic directed endosymbiosis
Source: Nat Commun. 2024 Jul 16;15:5947. doi: 10.1038/s41467-024-49585-3 (PMC11252298; doi:10.1038/s41467-024-49585-3)
Supplement: Supplementary file 3 — Description of Additional Supplementary Files [file 41467_2024_49585_MOESM3_ESM.pdf]

Description of Additional Supplementary Files:

File Name: Supplementary Data 1
Description: Sequences of double stranded DNA (gblocks) commercially obtained from Integrated DNA Technologies (IDT)

File Name: Supplementary Data 2
Description: Sequences of single stranded DNA oligonucleotides (primers) commercially obtained from Integrated DNA Technologies (IDT)
